# Migration of Bisphenol A and Its Derivatives From Epoxy Coatings and Demand for BPA‐NI Products: Scientific Insights and Perspectives Leading to Regulation (EU) 2024/3190

**DOI:** 10.1002/asia.202500340

**Published:** 2025-11-19

**Authors:** Takeru Kajiyama, Kohei Kanazaki, Gopinath Venkatraman, Puteri Shafinaz Abdul‐Rahman, Ayami Matsushima

**Affiliations:** ^1^ Laboratory of Structure‐Function Biochemistry Department of Chemistry, Faculty of Science Kyushu University Fukuoka Japan; ^2^ Universiti Malaya Centre For Proteomics Research Universiti Malaya Kuala Lumpur Malaysia; ^3^ Department of Biochemistry, Saveetha Dental College, Saveetha Institute of Medical & Technical Sciences Saveetha University Chennai India; ^4^ Department of Molecular Medicine Faculty of Medicine Universiti Malaya Kuala Lumpur Malaysia

**Keywords:** bisphenol A (BPA), bisphenol A‐non intent (BPA‐NI), endocrine‐disrupting chemical (EDC), epoxy resin, estrogen

## Abstract

Bisphenol A (BPA) is a widely used industrial chemical used in the production of polycarbonate and epoxy resins; however, it is also recognized as an endocrine‐disrupting chemical. This review examines scientific evidence on the migration of bisphenol A and its derivatives from epoxy can coatings into food products, highlighting the factors that have prompted recent regulatory shifts. The newly enacted Commission Regulation (EU) 2024/3190 addresses health concerns by promoting the use of BPA‐Non‐Intent (BPA‐NI) materials, which are formulated to avoid intentional BPA use and minimize contamination. This regulation is expected to significantly reduce the leaching of bisphenol A and its derivatives from epoxy resins into food and beverage cans, thereby enhancing consumer safety. This review also discusses key challenges and future directions for developing and evaluating BPA‐NI products and underscores the importance of continued research and innovation in this field.

## Graphical abstract

The newly enacted Commission Regulation (EU) 2024/3190 addresses health concerns by promoting the use of BPA‐Non‐Intent (BPA‐NI) materials, which are formulated to avoid intentional BPA‐related chemical use and minimize contamination.

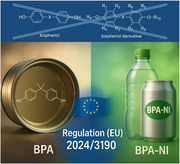

## 1 Introduction

**FIGURE 1:**
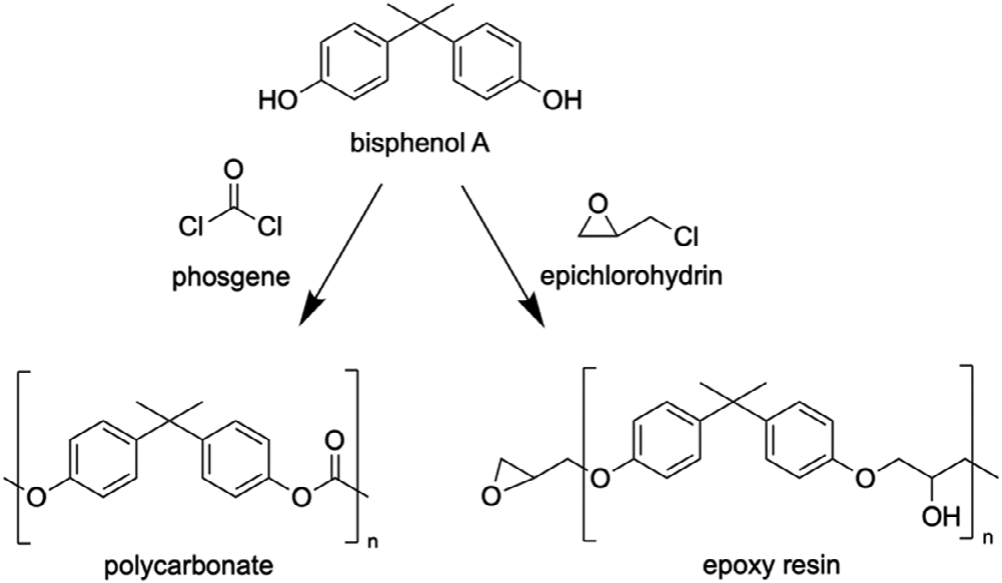
Polycarbonate and epoxy resins are synthesized from BPA.

Bisphenol A (BPA) is an industrial raw material known as a monomer for polycarbonate and epoxy resins (Figure 1). The official IUPAC name of this compound is 2,2‐bis(4‐hydroxyphenyl)propane (CAS Registry Number:80‐05‐7), with a molecular weight of 228.29 g/mol. Although it is produced and used globally, it is also recognized as a toxic environmental contaminant known as an endocrine‐disrupting chemical (EDC). BPA was first synthesized in the 1890s. In 1936, a report indicated that it demonstrated estrogenic properties despite lacking a steroid structure [1]. Although BPA exhibits estrogen‐like properties, its binding affinity for estrogen receptors is significantly lower, ranging from 1/1000 to 1/10,000 that of estradiol, the primary endogenous female hormone [2, 3]. Therefore, safety standards have been established; however, many studies have described the adverse effects of BPA [4, 5, 6, 7, 8]. In the late 1990s, scientists discovered that BPA exposure at levels near regulatory limits caused adverse effects in experimental animals such as rats and mice. These negative outcomes were subsequently categorized as low‐dose effects in this context [2, 9, 10]. However, the adverse effects largely depended on the experimental conditions, such as the species of animals used and individual differences. Numerous scientific studies have documented various phenomena, including prostate enlargement, its connection to cancer, variations in gene expression, and abnormal behavioral patterns [11, 12]. Conversely, some studies have suggested that no statistically significant negative effects are observed in animal experiments [13, 14]. Given these current circumstances, numerous countries are conducting safety assessments and research on bisphenol A. The United States initiated a comprehensive and extensive program in 2010 called the Consortium Linking Academic and Regulatory Insights on the Toxicity of BPA (CLARITY‐BPA). Currently, a compilation of the results from this initiative is available [15]. Despite extensive studies conducted to examine the harmful effects of BPA, the molecular pathways through which BPA negatively affects animals remain poorly understood.

There have been many reports of bisphenol A being detected in human blood [16], urine [17], and even breast milk [18, 19], and it is clear that the body absorbs bisphenol A released intentionally or unintentionally into the environment. Although establishing direct causal relationships through epidemiological research is challenging, the increasing body of human studies linking environmental BPA exposure to adverse health outcomes, combined with laboratory investigations across various species, including primates, provides growing evidence that environmental BPA exposure may be detrimental to human health. This is particularly concerning for behavioral issues and other effects observed in children [16]. This led to a preference for BPA‐free products that do not contain BPA. However, BPA‐free products often use raw materials that are not BPA, but contain similar chemical structures, which raises new safety concerns. It is unclear what is used as BPA‐free, but many BPA derivatives have been detected in samples of human origin, including breast milk. Many review papers have summarized BPA derivatives detected in human samples [18, 20, 21, 22, 23]. For example, 9,9‐bis(4‐hydroxyphenyl)‐fluorene (BHPF) eluted from BPA‐free plastic bottles showed an anti‐estrogenic effect, and the expression of estrogen‐responsive genes was reduced in animal studies using mice [24]. One of the interesting aspects of environmental chemical effects is the presence of gender differences in their effects, which has been explored in numerous articles [25, 26, 27]. Bisphenol A (BPA) exposure exhibits notable differences across various human population groups, particularly between infants and adults, due to variations in intake, metabolic capacity, and behavioral patterns [25]. When rats were exposed to BPA during their neonatal stage, they showed a higher occurrence of prostate intraepithelial neoplasia after being subjected to extended estradiol and testosterone exposure during their adult life [28].

An initial in vivo study on BPA conducted by endocrinologists in 1997 revealed that when pregnant mice were administered BPA at a low dose of 2 µg/kg/day, it resulted in adverse reproductive outcomes for their male offspring. Subsequently, numerous studies have documented harmful effects in animals exposed to low doses of BPA [29]. Similarly, a recent study assessed the oral administration of BPA to the rats during gestation (from gestational Day 4 to 14) and their health impact. The results show that the BPA exposure during pregnancy alters placental nutrient transport, hormonal signaling, and epigenetic profiles, potentially leading to obesity in offspring [30]. For instance, Gao et al. reported that BPA and BPS exposure (50 and 5000 µg/kg/day for 10 weeks) did not significantly affect glucose metabolism or Peroxisome proliferator‐activated receptor gamma (PPARγ) mRNA expression in the mouse liver. However, high doses of BPA and BPS significantly upregulated PPARγ target genes, such as *FABP4* and *LXRα*, indicating activation of the PPARγ pathway and altered liver metabolic profiles [31]. Furthermore, maternal BPA exposure in the female mouse model resulted in placental metabolic disturbances, increased expression of glucose transporter protein Glut1 and insulin‐like growth factor genes (*Igf1* and *Igfbp3*), and downregulation of key intermediates in glucose metabolism, including UDP‐D‐glucose, D‐glucosamine‐6‐phosphate, and glycolipid metabolism, in the placenta [32]. A recent study reported that low‐dose of BPA exposure (0.15 µg/L, a concentration commonly found in rivers) in the aquatic crustacean *Eriocheir sinensis* (Chinese mitten crab) leads to significant reproductive damage, including reduced testis weight and abnormal spermatogenic cell morphology. BPA acts as an estrogen analog, binding to the es‐ERR protein and disrupting the es‐ERR/es‐KIF4A pathway, which regulates spermatogenesis, leading to apoptosis and proliferation inhibition in spermatogenic cells [33].

Historically, the term “low‐dose” originally referred to exposure levels below 50 µg/kg/day, which was considered the lowest observed adverse effect level (LOAEL) based on studies available in the 1980s. However, this definition has evolved over time, there is no clear definition of low‐dose BPA; however, but in many cases, at concentrations lower than the official regulated levels, animal studies have indicated adverse effects of BPA [34]. Although these might not be direct reasons, the regulated level of BPA has been reviewed and redefined in the EU. Since products containing BPA are imported and exported worldwide, this is not a matter of regulation in a restricted region. In 2025, regulations regarding BPA and similar compounds were updated to Commission Regulation (EU) 2024/3190 in the EU. This recently enacted legislation amends Regulation (EU) No 10/2011 concerning plastics in contact with food and nullifies Regulation (EU) 2018/213 regarding the application of BPA in coatings and varnishes. Epoxy resins made from bisphenol and its derivatives have long been used to coat the inner surfaces of cans. As a result, they often come into contact with food in a tactile manner, and to meet this regulation, epoxy resins themselves must not be used. Recently, more BPA‐NI products have been used than BPA‐free products. The term “BPA‐NI” refers to “Bisphenol A Non‐Intent,” signifying that a product is not deliberately manufactured with BPA and its derivatives as ingredients. Nevertheless, it is crucial to understand that, despite the absence of intentional BPA addition, minute quantities might still be detected. This potential presence could result from unintended contamination during production or from the use of raw materials and packaging components. This review summarizes recent studies that have examined the leaching of BPA and its derivatives from epoxy resins in food or beverage cans to food products. The summarized evidence is intended to shed light on the scientific background that led to the enactment of Commission Regulation (EU) 2024/3190. It is expected that, after the regulation comes into effect, the release of BPA and its derivatives from epoxy resins in contact with food in cans will be greatly reduced.

## 2 BPA and Epoxy Resin and Its Applications

Many literature have mentioned that a Russian chemist, Aleksandr Dianin, was the first to synthesize BPA. He reported the synthesis of BPA as a synthesis of dimethyldiphenolmethane in the Journal of the Russian Physical Chemistry Society, published in 1891 under the title Condensation Products of Ketones and Phenols [35]. Indeed, the chemical structure of bisphenol A was illustrated on page 492 in the journal, which was written in Russian. Another mention of the synthesis of bisphenol A in an academic science journal was by Thomas Zincke in Germany in 1905 [36]. The synthesis of BPA is described in detail, but this study aimed to investigate the addition of bromine and chlorine to the aromatic rings of BPA. BPA is produced worldwide as a byproduct of polycarbonate and epoxy resin manufacturing; however, the exact amount of BPA production seems to be challenging to determine because many companies keep the actual amount of BPA production handled under trade secrets. For example, the United States Environmental Protection Agency (EPA) published Chemical Data Reporting (CDR) data [37]. Companies that produce or import specific chemicals to the United States, listed in the Toxic Substances Control Act (TSCA) Chemical Substance Inventory, must submit data to the EPA every 4 years if they reach certain yearly limits. This requirement typically applies when a single location produces or imports at least 25,000 pounds of chemical annually. The EPA implements rigorous data quality control measures to verify that the submitted information is accurately represented and that data designated as confidential business information (CBI) remain protected from disclosure or inference through data aggregation. The most recent publicly available data are the CDR 2020, which was disclosed in April 2022. The CDR2020 was based on the manufacturing outputs of 2016, 2017, 2018, and 2019. The CDR2020 data indicated that 34 companies manufactured or imported BPA, and 23 of them mentioned that the amounts were CBI. The total amount of BPA in the United States was estimated to be 1,000,000,000–5,000,000,000 lbs by the EPA.

The book “Our Stolen Future,” published in 1996 by Shea Colborn and colleagues, brought BPA to public attention. This book investigates the scientific evidence on EDCs, particularly how synthetic chemicals in the environment can interfere with hormone systems in humans and wildlife. The book was based on scientific papers. A previous study indicated that BPA leached from polycarbonate resin flasks subjected to autoclaving, subsequently promoting cell growth in MCF‐7 cells, an established breast cancer cell line. This effect mimicked that of estrogen [38]. Autoclaving is a sterilization method that employs high‐pressure saturated steam, typically at 120°C for 20 min. EDCs were originally named based on putative mechanisms that disrupt the natural endocrine system. Nevertheless, owing to increasing reports of negative impacts not only on reproductive systems but also on the central nervous system, EDCs are now also referred to as “hazardous (environmental) chemicals.”

BPA derivatives have been utilized as substitutes for BPA [39]; however, most BPA derivatives have not been developed as substitutes for BPA but as novel chemicals or raw materials for high‐performance resins. For instance, bisphenol S (BPS), 4,4′‐sulfonyldiphenol (CAS Registry Number 80‐09‐1), was first synthesized in 1867 by Ludwig Glutz, who referred to it as “oxysulphobenzide” [40]. BPS has often been used as a substitute for BPA in the production of thermal paper, and its use is restricted or prohibited in specific applications and regions, particularly in food contact materials and thermal paper [41]. A recent study reported the molecular mechanisms underlying the harmful effects of BPS [42, 43]. BPS primarily concentrates on the cellular membrane, where it interacts with a follicle‐stimulating hormone receptor [42]. This interaction triggers the activation of the downstream cyclic adenosine monophosphate/protein kinase A (cAMP/PKA) signaling cascade, ultimately resulting in the increased transformation of testosterone to 17β‐estradiol [42]. Bisphenol F (BPF), 4,4′‐bis (hydroxyphenyl)methane (CAS Registry Number 620‐92‐8), bisphenol AF (BPAF), and 2,2‐(4‐Hydroxyphenyl)hexafluoropropane (CAS Registry Number 1478‐61‐1) are well‐known BPA substitutes. Among BPA derivatives, bisphenol C (BPC) binds most strongly to estrogen receptor α [44, 45, 46]. BPC, 1,1‐dichloro‐2,2‐bis(4‐hydroxyphenyl)ethylene (CAS Registry Number 14868‐03‐2), was first synthesized in 1968 as a low material for polymer production. It should be noted that another compound has the same common name as “bisphenol C.” 2,2‐Bis(4‐hydroxy‐3‐methylphenyl)propane (CAS Registry Number 79‐97‐0) was also synthesized as a monomer for polymer production, and it is important to examine the chemical structure and/or CAS Registry Number when searching for papers. BPC (CAS Registry Number 14868‐03‐2) contains two chlorine atoms, and BPAF has six fluorine atoms. These compounds exhibit different activities than BPA, showing antagonistic activity against estrogen receptors [44, 45]. Several crystal structures of BPA and its derivatives bound to nuclear receptors have been reported, including BPA bound to estrogen receptor α (ERα)(PDB ID: 3UU7 [45]) and estrogen‐related receptor γ (ERRγ) (PDB ID: 2E2R [47], 2P7G [48], 6I63 [49]). For the crystal structures of the complexes of ERα and BPA derivatives, BPC (PDB ID: 3UUC [45]) and BPAF (PDB ID: 3UUA [45]) have been reported. No crystal structures have been reported for ERβ and BPA derivatives. The crystal structures of BPA derivatives bound to ERRγ have been reported for BPB (PDB ID: 6I61 [49]), BPE (PDB ID: 6I64 [49]), and several other BPA halogen adducts (PDB ID: 6K3N [50]). The structure of the Y215H mutated estrogen‐related receptor β (ERRβ) and BPA complex (PDB ID: 6LIT [51]) has recently been deposited. The structures of BPA derivatives bound to peroxisome proliferator‐activated receptor γ (PPARγ) have been deposited as BPB (PDB ID: 9F7X [52]), tetrabromobisphenol A (TBBPA) (PDB ID: 3OSW [53]), tetrachloro‐bisphenol A (TCBPA) (PDB ID: 3OSI [53]), and a TBBPA metabolite, monosulfate tetrabromobisphenol A (MonoTBBPA) (PDB ID: 3PBA [54]). The crystal structures of complexes [55, 56] of BPA derivatives and proteins are summarized in Table 1. These crystal structure data demonstrate direct binding of BPA and its derivatives to biological receptors.

**TABLE 1:** Crystal structures of complexes of BPA or its derivatives and proteins.

Protein	Category	BPA or BPA derivatives	PDB ID	References
Estrogen receptor α (ERα)	Nuclear receptor	BPA	3UU7	[45]
		BPC	3UUC	[45]
		BPAF	3UUA	[45]
Estrogen‐related receptor β (ERRβ), mutated(Y215H)	Nuclear receptor	BPA	6LIT	[51]
Estrogen‐related receptor γ (ERRγ)	Nuclear receptor	BPA	2E2R	[47]
		BPA	2P7G	[48]
		BPA	6I63	[49]
		BPB	6I61	[49]
		BPE	6I64	[49]
		Florinated BPA	6K3N	[50]
proliferator‐activated receptor γ (PPARγ)	Nuclear receptor	BPB	9F7X	[52]
		TBBPA	3OSW	[53]
		TCBPA	3OSI	[53]
		Monosulfate tetrabromobisphenol A	3PBA	[54]
dehaloperoxidase B	Enzyme	BPE	8DOG	[55]
		BPF	8DOH	[54]
Transthyretin	Transporter	BPS	5L4J	[56]

Polycarbonate and epoxy resins are manufactured using BPA and its related compounds as the primary ingredients. Epoxy resins constitute a crucial group of polymeric compounds defined by the presence of multiple three‐atom rings [57]. These cyclic structures are known by various names, including epoxy, epoxide, oxirane, and ethoxyline groups, and are fundamental to the compositions of these materials. The epoxy has its linguistic roots in Greek, combining “ep,” a prefix denoting “over and between,” with “oxy,” which relates to oxygen [57]. Epoxy resin is commonly synthesized through the reaction of epichlorohydrin with bisphenols such as bisphenol BPA or BPF. Owing to their excellent mechanical properties, chemical resistance, and strong adhesion, these resins are widely used as coatings, adhesives, composites, and sealants. The history of epoxy resins began with several patents [57]. A patent application for the synthesis of amine‐epoxy reaction products was submitted by Schlack from I.G. Farbenindustrie AG, a German company, in 1934. This application included a specific epoxy compound derived from bisphenol A and epichlorohydrin [58]. The industrial potential of epoxy resins has remained unrecognized for several years. Two companies operating independently in different countries have made this discovery simultaneously. The De Trey Freres Co. in Switzerland [59] and the DeVoe and Raynolds Co. [60]. The United States was the first to identify these commercial applications. In 1936, a scientist named Pierre Castan, working at the De Trey Freres Co., created a novel epoxy resin. This resin, which had a low melting point, was synthesized using BPA and epichlorohydrin. Ciba AG, a company based in Basel, Switzerland, has acquired licensing rights for patents. In 1946, the debut of the first epoxy adhesive occurred at the Swiss Industries Fair, where samples of casting resin were also made available to companies in the electrical sector [53]. Three‐quarters of the epoxy resins currently used worldwide are derived from bisphenol A diglycidyl ether (BADGE) [57, 61]. This compound is also called diglycidyl ether of bisphenol A (DGEBA). The prevalence of BPA epoxy resins stems from their cost‐effectiveness and ability to deliver satisfactory to superior results across numerous applications. Epoxy resin is used as an inner coating for food and beverage cans because of its excellent protective and functional properties, including adhesion strength, prevention of metal corrosion, and flexibility. However, during processing and storage, their derivatives can migrate into the food, potentially causing adverse health effects due to their genotoxic, mutagenic, and endocrine‐disrupting properties [62]. A recent study reported that the BADGE from epoxy‐coated cans specifically binds with amino acid residues such as cysteine (Cys), lysine (Lys), histidine (His), and glutamic acid (Glu). BADGE has electrophilic ethylene oxide rings that react with nucleophilic groups in these amino acids. The binding involves covalent modifications and is facilitated by hydrophobic interactions and hydrogen bonding. Moreover, the study highlights the potential for BADGE to alter protein structure and function, thereby posing safety risks, particularly in canned foods and supplemental proteins [63]. Additionally, Cyclodi‐BADGE (CdB), a chemical compound found in epoxy‐based coatings used in canned food packaging, poses potential health risks due to its high toxicity classification (Cramer Class III) [64]. Therefore, there is concern regarding the leaching of BPA and its derivatives into food and beverages, and many reports have directly measured their concentration. This is due to advances in technology that enable the detection of such chemicals. The analytical methods used to identify potential migrants from can coatings to food have already been reviewed comprehensively [19, 65]. Vealan and Chinnathambi developed a novel electrochemical sensor using La2Cu2O5‐modified glassy carbon electrodes (GCEs) for the rapid and sensitive detection of BPA in food‐contacting materials. The La2Cu2O5‐modified electrode exhibits high electrocatalytic activity, which enhances electron transfer kinetics. The sensor operates in phosphate‐buffered saline (PBS) at pH 7.0, where BPA undergoes electrochemical oxidation. The oxidation peak of BPA is then measured using square wave voltammetry (SWV), which is directly proportional to its concentration [66]. Similarly, a highly sensitive electrochemical sensor was developed using a nitrogen‐doped activated carbon derived from sweet potato biowaste (NSC‐600), which was blended with graphite powder and paraffin oil to create a modified carbon paste electrode (NSC‐600/CPE). Furthermore, BPA was detected through a diffusion‐controlled oxidation process, which transfers equal numbers of protons and electrons. The sensor employed SWV and linear sweep voltammetry (LSV) for BPA detection [67].

## 3 Leaching of BPA and Its Derivatives From Epoxy Resin in Cans

**FIGURE 2:**
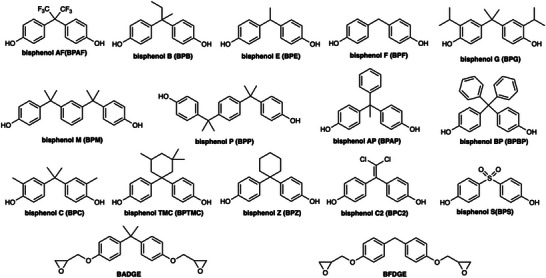
Structures of BPA and its derivatives analyzed in Table 2.

Many studies on BPA have been reported and have already been summarized in various review articles. A Scopus search using the keyword “bisphenol” review shows that a total of 1643 review articles have been published from 1970 to 2025, including 772 reviews during only 5 years from 2020 to 2025. To date, numerous reports have been published on BPA concentrations in the environment [68, 69, 70, 71, 72, 73, 74] and various beverages [75]. Table 2 summarizes the recently reported concentrations of BPA and its derivatives that are chemically transferred directly from epoxy resin in cans to food products or liquids. The chemical structures of analyzed BPA derivatives are shown in Figure 2. The mechanism of BPA leaching from food‐grade plastics involves the migration of BPA, an additive used in the manufacturing of polycarbonate plastics and epoxy resins, into food or beverages that come into contact with the plastic. Elevated temperatures could be one of the influential factors that weaken chemical bonds in the plastic structure, thereby increasing BPA leaching [76]. While the migration level varies depending on the packaging material, the can coatings exhibit a higher level of migration compared to plastic [77]. For instance, BPA leaching was observed from a polycarbonate drinking water container, which is linked to stressors such as UV exposure and elevated temperatures. ​Results showed that, after 12 days of UV exposure at approximately 30°C, BPA was detected at a level of 1.7 µg/L in the polycarbonate container [78]. Furthermore, the migration of BPA from plastic packaging into animal feed has been identified due to prolonged contact between the two [79]. These BPA releases are expected to be eliminated in the EU and in countries that import or export canned food or beverages to the EU as a result of the effective Regulation (EU) 2024/3190.

**TABLE 2:** BPA and its derivatives from epoxy resin in food or beverage cans.

Sample	Country of origin	Analyzed compounds	Published year	Method	References
Beverage	17 countries (purchased in Australia)	BPA (3.9–19,300 ng/L), BPAF (0.38–3.8 ng/L), BPB (21–35 ng/L), BPE (3.2 ng/L), BPF (6.3–5600 ng/L), BPG (107 ng/L), BPM (0.9–1.6 ng/L), BPP (9.6–143 ng/L), BPS (0.1–75 ng/L)	2024	LC‐MS/MS	[80]
Canned food (fruits and vegetables)	(Purchased in Iran)	BPA (1.62–21.87 ng/g)	2024	GS‐MS	[81]
Canned food and beverage (carbonated beverages and tuna; 86 samples)	(Purchased in Belgian)	BPA (average, 0.4 ng/g for beverage, 22.2 ng/g for tune); BPAF (average, 0.2 ng/g for beverage, 0.7 ng/g for tune); BPB (n.d.); BPE (n.d.); BPAP; BPBP; BPC; BPFL; BPTMC (0.1–3.6 ng/g)	2024	LC‐MS/MS	[82]
Canned food (130 samples)	(Purchased in Egypt)	BPA (5.75–710.59 ng/g)	2023	GS‐MS/MS	[83]
Canned see food (102 samples)	(Purchased in China)	BPA (n.d.–15.54 ng/g), BPF (N.D.–5.55 ng/g), BADGE (0.27–49.3 ng/g), BFDGE (0.53–4.66 ng/g)	2022	LC‐FLD	[84]
Resin	–	BPA (18 ng/g, water)	2021	FTIR	[85]
Resin	–	BPA (66.0–92.1 ng/g)	2021	LC‐MS/MS	[86]
Canned beverage	(Purchased in Slovenia)	Simulants were used. BPA, BPAF, BPF, BPE, BPC, BPB, BPC2, BPZ, BPS, BPAP	2020	LC‐DAD/MS	[87]
Food (meat products, aquatic foods, dairy products, edible oil, eggs, fruits, vegetables, and cereals)	(Purchased in Southwest Nigeria)	BPA (n.d.–28.4 ng/g)	2019	GS‐MS	[88]
Canned beverage (energy drink or coke)	Provide from supplier	BPA (0.99–9.40 ng/g)	2019	LC‐MS	[89]
Canned food (35 samples)	(Purchased in Lebanon)	BPA (average 0.50–109 ng/g)	2018	LC‐MS	[90]
Canned beverage (cola, diet cola, ginger ale, tea)	(Purchased in Canada)	BPA (0.085–0.32 ng/g), BPAF (n.d.), BPF (n.d.), BPB (n.d.), BPE (n.d.), BPB (n.d.)	2018	GC‐MS	[91]
Water pipe (drinking water)	(Purchased in Finland)	BPA (n.d.–23.5 ng/g)	2016	ICP‐MS	[92]
Canned food (fruit, fish, meat, tea, coffee; 78 samples)	(Purchased in Ottawa)	BPA (n.d.–54.56 ng/g)	2016	GC‐MS	[93]
Canned bear	(Purchased in China)	BPA (2.85 ng/g), BADGE (0.38 ng/g)	2015	LC‐FLD	[94]
Canned food (vegetables, fruit, fish, fruit juice)	(Purchased in USA)	BPA (0.26–19.83 ng/g)	2015	GC‐MS	[95]
Canned food (fish, meat, vegetable, fruit, other cooked foods, coffee, tea, and other beverages), (BPA‐reduced cans were used in Japan)	Japan, Italy, Mexico, Thailand, USA, China, Spain, Germany,	BPA (<5–390 ng/g)	2014	GC‐MS	[96]
Water bottles	(Purchased in China)	BPA (2.89–90.4. ng/g)	2014	ICP‐MS	[97]
Water pipe (drinking water)	France, UK, USA, Spain	BPA (n.d.), BPF (n.d.)	2014	GC‐MS	[98]
Food (including canned food; 289 food samples)	(Purchased in China)	BPA (n.d.–59.6 ng/g), BPAF (n.d.–0.505 ng/g), BPAP (n.d.–127 ng/g), BPB (n.d.), BPF (n.d.–263 ng/g), BPP (n.d.–73.1 ng/g), BPS (n.d.–24.8 ng/g), BPZ (n.d.–0.401 ng/g)	2014	LC‐MS/MS	[99]
Canned food (vegetables, fruit, fish, meat, paghetti, chili; 80 samples)	(Purchased in Washington, DC)	BPA (2.6–760 ng/g)	2011	LC‐MS/MS	[100]
Canned food (beverage, fruit)	Portugal, Indonesia, Thailand, Kenia, Spain, China, Italy, France, EU	BPA (n.d.–265.6 ng/g), BPB (n.d.–3.4 ng/g)	2013	GC‐MS	[101]
Canned food	(Purchased in USA)	BPA (<0.02–65.0 ng/g)	2010	GC‐MS	[102]
Canned food and beverage (45 samples)	(Purchased in Belgian)	BPA (n.d.–8.1 ng/g)	2010	GC‐MS	[103]
Canned food (fruit, fish, meat, tea, coffee; 62 samples)	(Purchased in Korea)	BPA (n.d.–54.56 ng/g)	2009	LC‐MS	[104]
Canned tomato	Italy, China	BPA, BPB, BADGE	2008	LC‐MS	[105]
Canned food (fish)	Unknown	BPA (11.3–138.4 ng/g)	2005	LC‐MS	[106]
Canned food (vegetables, fruit, fish, soup and sauces, meat, paghetti, infant food, beverages)	NZ, Australia, Italy, Thailand, South Africa, Philippines, Canada, Alaska, 80 samples (purchased in Christchurch)	BPA (<10–109 ng/g)	2005	LC‐MS	[107]
Resin	–	BPA (n.d.–237.4 ng/g)	2002	GC‐FID	[108]
Canned food (fish, vegetables, soup, desserts, fruit, infant food, pasta, meat; 62 samples)	(Purchased in UK)	BPA (<5–95.3 ng/g), BPF (n.d.)	2002	LC	[109]
Canned food (fruit, vegetables)	USA, Japan, Indonesia, China, Australia, Thailand (purchased in Japan)	BPA (<5–95.3 ng/g)	2001	LC	[110]
Canned food (fish, meet, infant food, cola)	(Purchased in the USA)	Diglycidyl ether of bisphenol A (BADGE) (n.d.–50 µg/g, extracted)	1999	LC	[111]

The detection limits in reported studies on BPA vary significantly depending on the analytical method employed, the specific matrix being analyzed, and the overall rigor of the study's quality control [39, 112]. For example, the detection limit for BPA was 0.1–0.7 µg/kg in carbonated beverages and canned tuna using LC‐MS/MS, and this was slightly higher than Immunoaffinity Chromatography studies (0.007–0.05 µg/kg) but similar to solid phase extraction cartridge methods (0.01–20 µg/kg) [82]. The complexity of food and biological matrices can significantly influence analytical performance due to “matrix effects,” which can suppress or enhance the analyte signal [39]. This necessitates robust sample preparation techniques like solid phase extraction or liquid‐liquid extraction [39, 91]. However, even these can have limitations, such as reduced recovery in complex matrices or interference from background contamination [39, 91, 112].

Quantifying Bisphenol A (BPA) and its analogues in various matrices, such as food, beverages, and biological samples, rely on a diverse array of analytical methods. These methods typically involve sample preparation and extraction techniques, followed by instrumental detection and quantification. For example, liquid–liquid extraction (LLE) is a relatively simple method that uses solvents to extract the analyte from the sample [19, 82, 112]. While effective for various biological fluids (urine, plasma, serum, amniotic fluid), the method is often time‐consuming and requires large volumes of organic solvents, and can be difficult to automate [112]. Challenges include finding optimal solvents for polar bisphenol analogues and potential emulsion formation [112]. Solid‐phase extraction (SPE) is widely used technique involves passing a liquid sample through a solid adsorbent that selectively retains target analytes, allowing for concentration and purification [19, 74, 82, 112]. It offers better selectivity and higher recoveries than conventional LLE [112]. SPE is the most commonly employed technique for liquid biological samples, with both offline and online variations [112]. However, its selectivity can be limited, potentially leading to poor recovery from complex matrices or clogging of columns [82]. QuEChERS (Quick, Easy, Cheap, Effective, Rugged, and Safe) is two‐step method involves an extraction step based on salting‐out partitioning (often with acetonitrile) and a clean‐up step with dispersive SPE (d‐SPE) [19, 112]. It is frequently used for breast milk due to its effectiveness and simplicity [112]. As for analytical methods, chromatography and mass spectrometry‐based techniques are the most common analytical approaches for BPA detection, offering high sensitivity and specificity, which are crucial given the low concentrations of bisphenols in samples [112], as indicated in Table 2. Combinations of liquid chromatography and mass spectrometry (LC‐MS or LC‐MS/MS) are the sensitive and widely used method for BPA detection in human urine and biological matrices [80, 82, 86, 89, 90, 99, 100, 104, 105, 106, 107]. It provides high sensitivity, specificity, and rapid analytical capabilities, even in challenging matrices, by maintaining low limits of detection. Combinations of gas chromatography and mass spectrometry (GC‐MS or GC‐MS/MS) are frequently used and highly sensitive methods for quantifying bisphenol analogues, offering high molecular specificity [81, 83, 88, 91, 93, 95 ^,^ 96, 98, 101, 102, 103]. A disadvantage is that BPA analysis often requires a derivatization step (e.g., acetylation or silylation with reagents like MSTFA, BSTFA, PFBBr, acetic anhydride, or TBAOH) to improve peak shapes, volatility, and robustness for GC [19]. LC with fluorescence detection (LC‐FLD) is a sensitive, inexpensive, and valuable technique, capable of detecting BPA at parts per billion levels without derivatization, and generally offers lower limits of detection compared to UV detection [112].

Interestingly, these reports show that leaching from food cans was low in New Zealand, Korea, and Japan [96, 104, 107]. The decreased exposure to BPA in Japan is likely attributed to the widespread adoption of “BPA‐reduced cans” in Japan, which were developed by Japanese can manufacturers in the late 1990s [96]. BPA‐reduced cans were produced by controlling the heating conditions during the coating of the epoxy resin. Another putative reason seemed to be the development and merchandising of a groundbreaking technique known as Toyo Ultimate Laminate Cans (TULC) by a Japanese company called Toyo Seikan Co. in the 1980s [57, 113]. This innovative process involves creating cans through deep drawing, using metal coils that have been coated with thermoplastic polyester films; however, the expenses are considerably higher than those of traditional cans, and this technology has only been used in Japan [57]. In a recent development in the United States, Campbell Soup Co. achieved success with the introduction of a new product line featuring microwaveable, ready‐to‐eat containers made of plastic. These innovative containers consist of a can body formed from thermoplastic materials (specifically polypropylene and high‐density polyethylene) and incorporate a coated metal easy open end (EOE) [57].

## 4 Commission Regulation (EU) 2024/3190 and BPA‐NI Products

**FIGURE 3:**
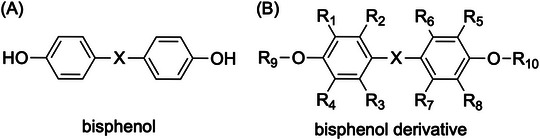
Bisphenol and bisphenol derivatives are regulated in Commission Regulation (EU) 2024/3190. The note of this regulation mentions “X refers to any bridging group separating the two phenyl rings by one single atom, but the atom can have any substituent(s). R1 to R10 refers to any substituent. At least one of the substituents is not a hydrogen atom (H).”

A concern with BPA and its derivatives is that low‐dose effects of BPA have been reported. There is no clear definition of “low‐dose,” but a paper mentions any dosages that fall below the previously established lowest observed adverse effect level (LOAEL) of 50,000 µg/kg/day, as determined by the Environmental Protection Agency (EPA) and Food and Drug Administration (FDA) [34]. The tolerable daily intake (TDI) was set at 0.2 ng/kg/day by the EFSA [114] in the EU and to 50 µg/kg/day by the U.S. Environmental Protection Agency (USEPA) in the United States [34]. These concerns have led the resin suppliers and can makers to create various bisphenol A non‐intent products, referred to as BPA‐NI products. A new, comprehensive regulation concerning the usage of bisphenol A (BPA) and related compounds in specific food contact materials (FCM) and products has been introduced by the European Union (EU) [115]. The Regulation (EU) 2024/3190 prohibits the use of BPA and its salts in the manufacture of various food contact materials and articles, including adhesives, rubbers, ion‐exchange resins, plastics, printing inks, silicones, and varnishes and coatings [115]. This entered into force on January 20, 2025. This recently enacted legislation amends Regulation (EU) No 10/2011 [116] concerning plastics in contact with food and nullifies Regulation (EU) 2018/213 [117] regarding the application of BPA in coatings and varnishes. It is noteworthy that the European Union has transitioned toward structure‐based regulations for bisphenols, moving beyond individual CAS (Chemical Abstracts Service) number‐based restrictions, primarily to address the phenomenon of “regrettable substitution” and to ensure comprehensive public health protection [115]. This regulation includes not only currently existing compounds but also those that may be synthesized in the future. Bisphenols and bisphenol derivatives restricted by this regulation are defined by their chemical structural formulas and not by their compound names (Figure 3). In comparison, previous regulations often indicated compounds using CAS Registry No. This regulation facilitated the use of BPA‐NI products, signifying that a product was not intentionally used with BPA and its derivatives as ingredients. The direct leaching of BPA and BPA derivatives from cans into food and beverages, as summarized in Table 2, will be reduced by replacing them with BPA‐NI products.

## 5 Summary and Outlook

**FIGURE 4:**
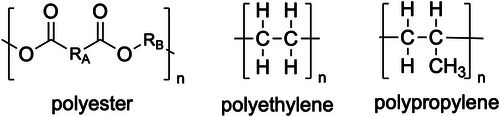
Chemical structures of representative BPA‐NI materials used in place of BPA‐based epoxy resins.

**FIGURE 5:**
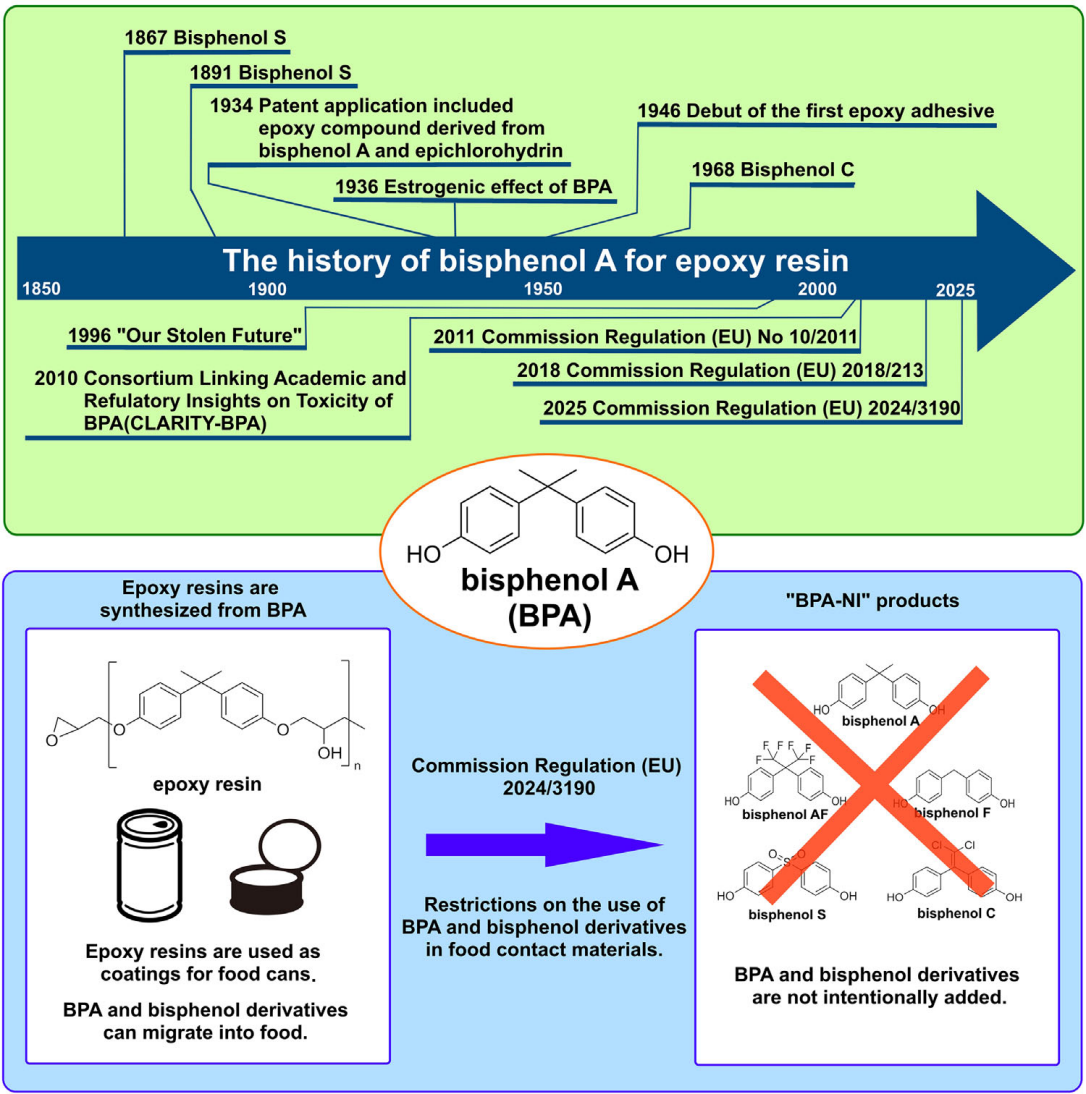
Summary of events related to BPA.

BPA is widely used as an industrial raw material for the production of polycarbonate and epoxy resins. However, its estrogenic effects were first reported in 1936, and low doses have been observed since the 1990s. Nevertheless, the mechanism underlying these low‐dose effects remains unclear. As a result, bisphenol derivatives came to be used as alternatives to bisphenol A; however, concerns have also been raised regarding the safety of these substitutes. Many bisphenol derivatives show much higher binding ability to estrogen receptor alpha and beta [44] and have been reported to exhibit higher or similar toxicity compared to BPA itself [12, 20, 22, 39]. Despite extensive research on BPA, numerous significant unknowns and knowledge gaps persist, particularly concerning its long‐term health effects, the molecular mechanism of the low‐dose effects, the impact of its substitutes, and the complex dynamics of human and environmental exposure at environmentally relevant levels [114]. In 2025, Commission Regulation (EU) 2024/3190 was enacted to regulate the use of BPA and its derivatives in food‐contact materials [115]. In this raw, BPA derivatives with harmonized classification for specific hazardous properties are largely regulated to a similar extent as BPA itself, with prohibitions on their use in many food contact materials and articles. The intent of this regulation is to prevent the “regrettable substitution” of BPA with other bisphenols that may pose similar risks [115]. As BPA‐NI materials, alternatives such as polyester, plant‐based coatings, polyethylene (PE), polypropylene (PP) (Figure 4), bioplastics, and glass are currently being used in place of BPA‐based epoxy resins [118]. Polyester‐based BPA‐NI coatings generally offer comparable corrosion resistance to traditional BPA‐based epoxies, though they may show reduced solvent and alkali resistance. Their thermal performance, particularly under pasteurization conditions, can be inferior; however, ongoing formulation improvements are addressing these limitations to ensure food and beverage safety [57]. Recently, a study thoroughly examined a BPA‐NI dual‐layer polyester‐phenol coating, which is widely employed in the commercial production of metal lids for sterilized glass containers of complementary infant food, and identified the leaching of cyclic polyester oligomers [119]. In this study, polyester‐based coatings, which are used as BPA non‐intent alternatives, have been shown to release cyclic polyester oligomers into food simulants and infant food [119]. This highlights that new, non‐BPA alternatives can introduce a different set of non‐intentionally added substances into food [119]. A previous study evaluated the stability of BPA‐NI using electrochemical impedance spectroscopy [120], and it is expected that further progress will be made in evaluating BPA‐NI cans. The Commission Regulation (EU) 2024/3190 is in force only in the EU; however, many countries outside the EU should also consider adapting similar legislation, as global import and export markets operate on an international scale. Although BPA contamination remains elusive, it is expected that the direct leaching of BPA and its derivatives from epoxy resins in cans will soon become virtually undetectable by the regulations. Its practical effectiveness in reducing trace contamination from sources other than food‐related products will become clearer through future compliance studies and surveillance data. Despite extensive research on BPA and its analogues, significant scientific uncertainties remain, particularly regarding their long‐term health effects, low‐dose mechanisms, and the behavior of emerging substitutes (Figure 5). To address these limitations, future studies should adopt a multidisciplinary approach combining advanced exposure monitoring, sensitive biological models, and rigorous analytical methods. Such efforts will be essential for accurately assessing health risks and supporting evidence‐based policy development aimed at protecting public health.

## Conflicts of Interest

The authors declare no conflicts of interest.

## Data Availability

Data sharing is not applicable to this article as no new data were created or analyzed in this study.
